# Population genetic structure and adaptive differentiation of iron walnut *Juglans regia* subsp. *sigillata* in southwestern China

**DOI:** 10.1002/ece3.5850

**Published:** 2019-11-21

**Authors:** Yi‐Wei Sun, Na Hou, Keith Woeste, Chuchu Zhang, Ming Yue, Xiao‐Ying Yuan, Peng Zhao

**Affiliations:** ^1^ Key Laboratory of Resource Biology and Biotechnology in Western China Ministry of Education College of Life Sciences Northwest University Xi'an China; ^2^ Guizhou Academy of Forestry Guiyang China; ^3^ Department of Forestry and Natural Resources USDA Forest Service Hardwood Tree Improvement and Regeneration Center (HTIRC) Purdue University West Lafayette IN USA; ^4^ Xi'an Botanical Garden of Shaanxi Province Xi'an China

**Keywords:** evolutionary history, gene flow, genetic differentiation, genetic structure, *Juglans sigillata*, Southwest China

## Abstract

Southwestern (SW) China is an area of active tectonism and erosion, yielding a dynamic, deeply eroded landscape that influences the genetic structure of the resident populations of plants and animals. Iron walnut (*Juglans regia* subsp. *sigillata*) is a deciduous tree species endemic to this region of China and cultivated there for its edible nuts. We sampled 36 iron walnut populations from locations throughout the species' range in SW China and genotyped a total of 765 individuals at five chloroplast DNA regions and 22 nuclear microsatellite loci. Species distribution models were produced to predict the evolution and historical biogeography of iron walnut and to estimate the impacts of climate oscillations and orographic environments on the species' demography. Our results indicated that *J. regia* subsp. *sigillata* had relatively low genetic diversity, high interpopulation genetic differentiation, and asymmetric interpopulation gene flow. Based on DIYABC analysis, we identified two lineages of *J*. *sigillata* in southwestern China. The lineages (subpopulations) diverge during the last glacial period (~1.34 Ma). Southwestern China was a glacial refuge during the last glacial period, but increasingly colder and arid climates might have fostered the fragmentation of *J*. *regia* subsp. *sigillata* within this refugium. Finally, we found that recent habitat fragmentation has led to a reduction in population connectivity and increased genetic differentiation by genetic drift in isolated populations. Our results support a conclusion that geological and climatic factors since the Miocene triggered the differentiation, evolutionary origin, and range shifts of *J*. *sigillata* in the studied region.

## Graphical abstract

Southwestern (SW) China is an area of active tectonism and erosion, yielding a dynamic, deeply eroded landscape that influences the genetic structure of the resident populations of plants and animals. Iron walnut (*Juglans sigillata* Dode) is an endemic, deciduous tree species native to this region of China and cultivated there for its edible nuts. We sampled 36 wild iron walnut populations from locations throughout the species' range in SW China and genotyped a total of 765 individuals at five chloroplast DNA regions and 22 nuclear microsatellite loci. The data were used to infer levels of genetic differentiation, genetic structure, and contemporary/historical levels of gene flow. Species distribution models were produced to predict the evolution and historical biogeography of iron walnut, and to estimate the impacts of climate oscillations and orographic environments on the species' demography.

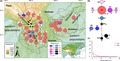

## 1 INTRODUCTION

Southwestern (SW) China is a unique alpine area with a range of climates, complex topography, and a high proportion of endemic and relict flora (about 29% of species). It is considered as a hot spot for vascular plant biodiversity (López‐Pujol, Zhang, Sun, Ying, & Ge, 2011; Myers, Mittermeier, Mittermeier, Da Fonseca, & Kent, 2000) and the core region of genetic diversity for many species (Brookfield, 2008). Of particular biogeographic interest are the Hengduan Mountains, which are located in the southeastern part of the Qinghai–Tibet Plateau (Du, Hou, Wang, Mao, & Hampe, 2017). This range includes mountains in Sichuan province, Yunnan province, and eastern Tibet Autonomous Region (Zhao, Gugger, Xia, & Li, 2016). The southern extreme of the Hengduan Mountain chain includes the Wuliang Mountains, which are located in northwestern Yunnan, the Yunnan–Guizhou plateau, and the convergence point of the Central and South Asian tropical zones (Myers et al., 2000). To examine the connections among geography and climatic oscillations and the processes that drive genetic differentiation, we examined the biogeography of the perennial, woody species iron walnut (*Juglans regia* subsp. *sigillata*).

With regard to the systematic status of *J. sigillata*, there is a long‐term, heated debate (Kuang & Lu, 1979; Wang, Pei, Gu, & Wang, 2008; Wu, Pei, Xi, & Li, 2000; Yang & Xi, 1989). It has been regarded as an ecotype of *J*. *regia*, but numerous botanists have viewed it as a different species (Aradhya, Potter, Gao, & Simon, 2007; Kuang & Lu, 1979). *Juglans* *sigillata* was identified as a new species by Dode, whose taxonomy was based on morphological differences *J*. *sigillata* individuals are distinguished from *J*. *regia* by their relatively large compound leaves, rugose trunks, thick endocarp, and deeply pitted nut surfaces (Dode, 1909a, 1909b). Yang and Xi (1989) also suggested that *J*. *sigillata* and *J*. *regia* are ecological types of one species based on the isoenzyme peroxidase (Yang & Xi, 1989). On the basis of RAPD markers, Wu et al. (2000) determined *J*. *sigillata* to be an independent species, and an analysis of ITS, RFLP, and cpDNA affirmed that *J*. *regia* and *J*. *sigillata* are distinct (Aradhya et al., 2007). In general, the most recent studies of *J*. *regia* and *J*. *sigillata* conclude they are a single species (Wang, Pan, Ma, Zhang, & Pei, 2015; Wang et al., 2008).

Transcriptome data generated by high‐throughput sequencing have been an excellent resource for SSR marker development (Dang et al., 2016; Hu et al., 2016). EST‐SSRs have a variety of uses; some sequences contain polymorphisms that are transferable to related taxa (Feng et al., 2018). Analysis of EST‐SSRs in populations where *J. regia* and *J. sigillata* are sympatric in SW China revealed that the *J*. *sigillata* and *J*. *regia* populations were divided into two genetic clusters with frequent gene introgression (Yuan, Zhang, Peng, & Ge, 2008), but we found that iron walnut should be considered a subspecies or landrace of *J. regia* based on genotype by sequencing and EST‐SSRs data (Feng et al., 2018). Whatever the status of *J. sigillata*, the evolutionary and ecological changes that led to the current genetic patterns remain understudied.

Biogeographical studies of angiosperms frequently combine nuclear markers, such as SSRs, which are inherited biparentally, with chloroplast markers that are inherited matrilineally. By comparing results from both types of markers across the same sampled populations, complementary views of a species' evolution, genetic structure, differentiation, and gene flow can be obtained (McCauley, 1995; Mohammad‐Panah, Shabanian, Khadivi, Rahmani, & Emami, 2017). Here, we use a multidisciplinary approach including molecular phylogeographic, ecological niche modeling, and phylogenetic approaches to investigate the biogeography, genetic structure, and demographic history of iron walnut in China. Specifically, we used 22 simple sequence repeat (EST‐SSR) markers and sequence variation at five chloroplast fragments (cpDNAs) to (a) determine the genetic diversity and population structure of *J*. *sigillata* at chloroplast DNA sequences and nuclear markers; (b) estimate the degree of population differentiation and gene flow of *J. sigillata* populations from the different regions; and (c) determine divergence times of two populations within *J*. *sigillata* and of *J*. *sigillata* from *J*. *regia*; (d) deduce current and past population and range dynamics [during the last interglacial (LIG; 130–116 kyr BP) and the last glacial maximum (LGM; 21–18 kyr BP)], and understand their possible underlying environmental causes.

## 2 MATERIALS AND METHODS

### 2.1 Plant sampling, DNA extraction, Microsatellite genotyping, and Chloroplast DNA sequencing

In 2015 and 2016, leaf samples of 765 *J. sigillata* were collected from 36 apparently autochthonous populations in SW China. Information of samples can be inferred from a material table provided at Dryad under https://doi.org/10.5061/dryad.h70rxwddv. Total genomic DNA was isolated from the dried leaf tissue using a Plant Genomic DNA extraction kit from TIANGEN (TIANGEN, Beijing, China). Genotypes of all DNA samples were detected using 22 pairs of microsatellite primers developed for *Juglans* (Dang et al., 2015, 2016; Hu et al., 2015, 2016). Each forward primer was marked with fluorescent dye (FAM, TAMRM, HEX, and ROX). The PCR products were detected using software GeneMarker (Holland & Parson, 2011). A subset of 186 individuals from 31 *J. sigillata* populations and 12 individuals from two *J. regia* populations, and three black walnut (*J. nigra*) individuals (used as an out‐group) were sequenced at five chloroplast DNA regions (Table S1). All chloroplast sequences were deposited in GenBank under accession numbers MH606007–MH606019.

### 2.2 Genetic diversity statistical analyses based on EST‐SSR data

A set of 765 individuals from 36 *J. sigillata* populations plus 12 *J. regia* populations (269 individuals) were evaluated at 22 EST‐SSRs. We calculated *F*_ST_ to identify outlier EST‐SSR loci as potentially under selection (Tsuda, Nakao, Ide, & Tsumura, 2015). Non‐neutrality of loci was detected using Arlequin version 3.5 (Excoffier & Lischer, 2010) (Figure S1). The genetic diversity parameters were computed by the GenALEx 6.5 (Peakall & Smouse, 2012). The Inverse Distance Weighted (IDW) of spatial interpolation analysis in the Geographic Information System (GIS) software ArcGIS 10.0 was implemented to display the geographic patterns of the number of alleles (*N*_A_), expected heterozygosity (*H*_E_), allelic richness (*R*_S_), genetic differentiation (*F*_ST_), and private allele richness (*P*_AR_) for 36 populations; then, we displayed the geographic patterns of the haplotypes based on cpSSR markers (Feng et al., 2018).

### 2.3 Haplotype network reconstruction and nucleotide diversity analysis

All the chloroplast sequence data were edited and aligned using BioEdit v. 7.2.5 (http://www.mbio.ncsu.edu/bioedit/bioedit.html). Haplotype diversity (*H*_d_) and nucleotide diversity (*π*) were estimated by DnaSP 5.0 (Rozas, Sánchez‐DelBarrio, Messeguer, & Rozas, 2003). We used Network 4.6.13 to generate minimum spanning tree using median‐joining (Bandelt, Forster, & Röhl, 1999). Mismatch distribution analyses (MDA), Tajima's *D* (Tajima, 1989), and Fu and Li's *F* (Fu & Li, 1993) were estimated using Arlequin v. 3.5 (Excoffier & Lischer, 2010).

### 2.4 Population structure analysis and population differentiation statistics

Genetic structure of 36 iron walnut populations was predicted using the software STRUCTURE version 2.3.4 (Evanno, Regnaut, & Goudet, 2005) based on 22 SSRs (12 neutral and 10 non‐neutral) in 36 iron walnut populations sampled across their native range. Principal component analysis (*PCoA*) was conducted after examining genetic distance with GenALEx 6.5 (Peakall & Smouse, 2012). Neighbor joining (NJ) based on Nei's genetic distance (Nei, 1972) was performed using POP TREE2 software (Takezaki, Nei, & Tamura, 2014) with the support of 1,000 arbitrary bootstraps. An analyses of molecular variance (AMOVA) were run with 1,000 permutations in Arlequin 3.5 (Excoffier & Lischer, 2010) based on microsatellites data and cpDNA sequence data.

### 2.5 Estimation of divergence time

BEAST v 1.8.0 was used to estimate phylogenetic relationships and divergence times between lineages (Drummond, Suchard, Xie, & Rambaut, 2012). We choose *J. nigra* as the out‐group. Calibration of the tree was based on fossil evidence, which indicates the time of divergence between *Juglans* sect. *Rhysocaryon* and sect. *Dioscsryon* was 45 Ma (Aradhya et al., 2007; Bai, Wang, & Zhang, 2016). We used the GTR + I+G nucleotide substitution model, an uncorrelated log‐normal clock, and a Yule process tree prior to estimate the divergence times of the main clades. Effective sample size (ESS) value was > 200. Bayesian skyline plots (BSP) were used to conclude variation in effective population size (*N*e) by BEAST v1.8.3 (Wang et al., 2017).

### 2.6 Inferring demographic history of *J. sigillata* using approximate Bayesian computations (ABC) analysis

We used DIYABC v. 2.0 software to infer recent colonization history using an approximate Bayesian computation algorithm (ABC) (Cornuet et al., 2014). Based upon previous studies (Tsuda et al., 2015), the generation time of walnuts was assumed to be 50 years, and we pooled subsets of *J. sigillata* samples into two subpopulations as inferred by STRUCTURE (Figure S2). All haplotypes of *J. sigillata* clustered into two clades (clade A and clade B). Gene pool I consisted of 377 individuals from 22 populations, and gene pool II consisted of 388 individuals from 14 populations. The *J*. *regia* population was considered gene pool III (Figure S3). We tested 13 competing broad‐scale scenarios, and total 13 million simulations were run for all scenarios (13 scenarios; Figure S4).

### 2.7 Historical gene flow, contemporary gene flow, and genetic barriers

To explore historical gene flow within *J*. *sigillata* populations, we employed MIGRATE v 3.6.1.1 (Beerli, 2006). All samples of *J. sigillata* were divided into two subpopulations and one of *J. regia* based on STRUCTURE analysis using SSR data. In order to ensure the veracity and consistency of our results, we ran Migrate five independent times. Genetic barriers were investigated using Monmonier's maximum difference algorithm as implemented in the software Barrier v. 2.2 (Manni, Guerard, & Heyer, 2004).

### 2.8 Species distribution modeling

Species distribution modeling for *J. sigillata* was carried out in MAXENT v. 3.3.3 (Feng et al., 2018; Phillips & Dudík, 2008). A total of 274 distribution records were retrieved from the Chinese Virtual Herbarium and National Specimen Information Infrastructure. Eight variables were discarded from models because they were highly correlated environmental variables (Pearson's correlation coefficient >0.85), leaving 11 bioclimatic variables. To determine whether the calculated niche similarity metrics were significant, we performed identity tests in ENMTOOLS (Warren, Glor, & Turelli, 2010).

## 3 RESULTS

### 3.1 Distribution of cpDNA haplotypes

**Figure 1:**
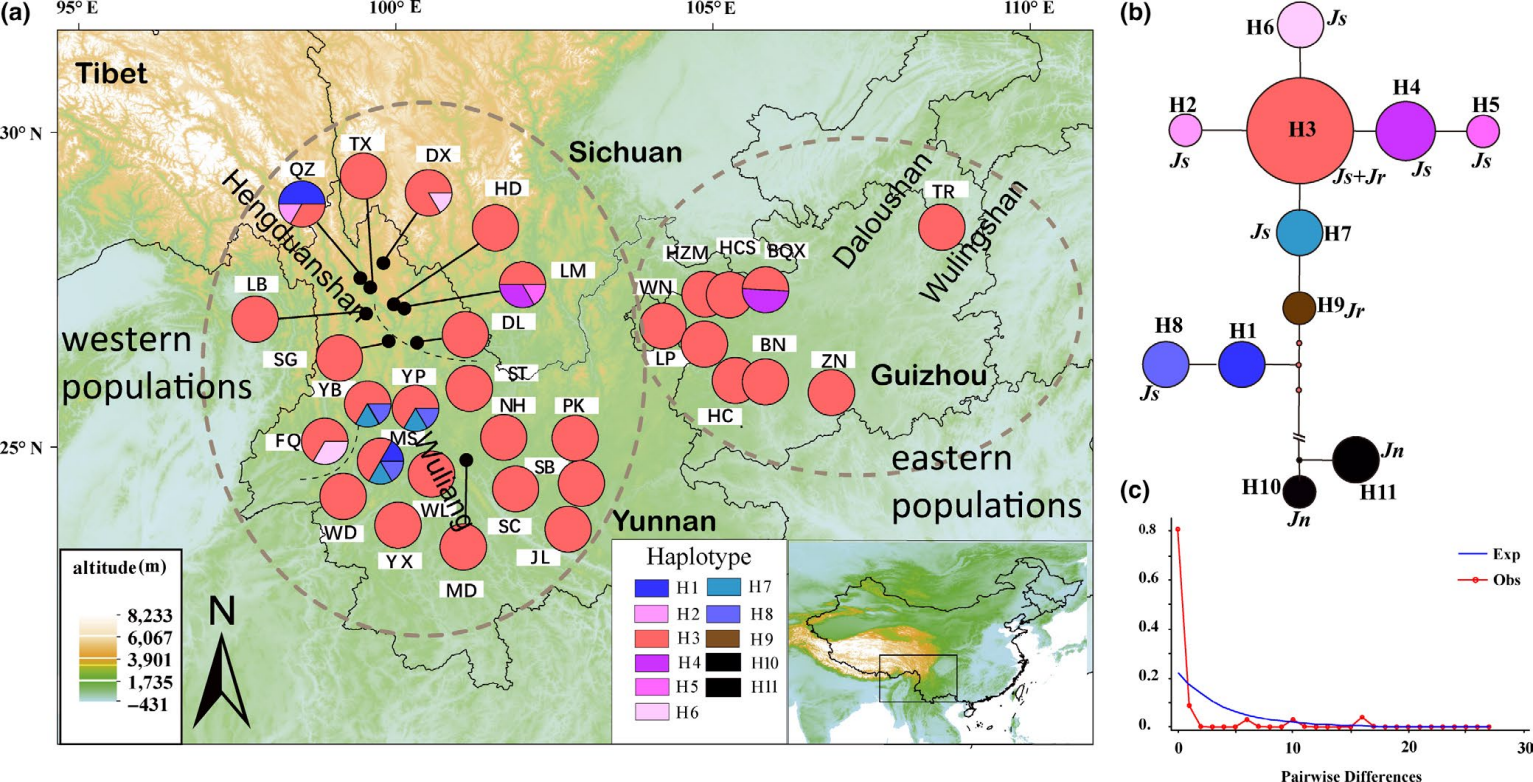
Geographical distribution and cpDNA structure of all chloroplast haplotypes of *Juglans sigillata*. (a) Geographical distribution of chloroplast haplotypes in the 31 populations. Haplotype colors correspond to those shown in the left panel (b) Inferred phylogenetic network of the 11 cpDNA haplotypes found. (c) Distribution of the number of pairwise nucleotide differences for cpDNA sequence data in *Juglans sigillata*. Js *J. sigillata*, Jr *J. regia*, Jn *J. nigra*

We observed 11 chloroplast haplotypes over a combined 1,597 bp based on five cpDNA fragments (Table S1). There were 28 variable sites: 18 substitutions and 10 indels. Across 31 populations of *J*. *sigillata*, haplotype diversity (*H*_d_) was 0.2026 and nucleotide diversity (*π*) was 0.0008. Most populations (74.2%) shared haplotype H3, while only eight populations out of 31 (25.8%) had two, three or four Cp haplotypes. Higher diversity was found in western (Wuliang Mountains; *H*_d_ = 0.259; *π* = 0.00125) populations (QZ, LM, YB, SM, and YP) versus eastern populations (*H*_d_ = 0.081; *π* = 0.00005) (Figure 1a; Table S1). Based on comparison with the out‐group *J*. *nigra* (H11 and H10), which differed by 236 mutation steps, haplotypes H1, H7, H8, and H9 were more ancient than H3, although H3 was the most widespread and most common. All individuals of *J*. *regia* showed haplotype H3 or H9 (Figure 1b).

### 3.2 EST‐SSR polymorphism and population' genetic diversity

**Figure 2:**
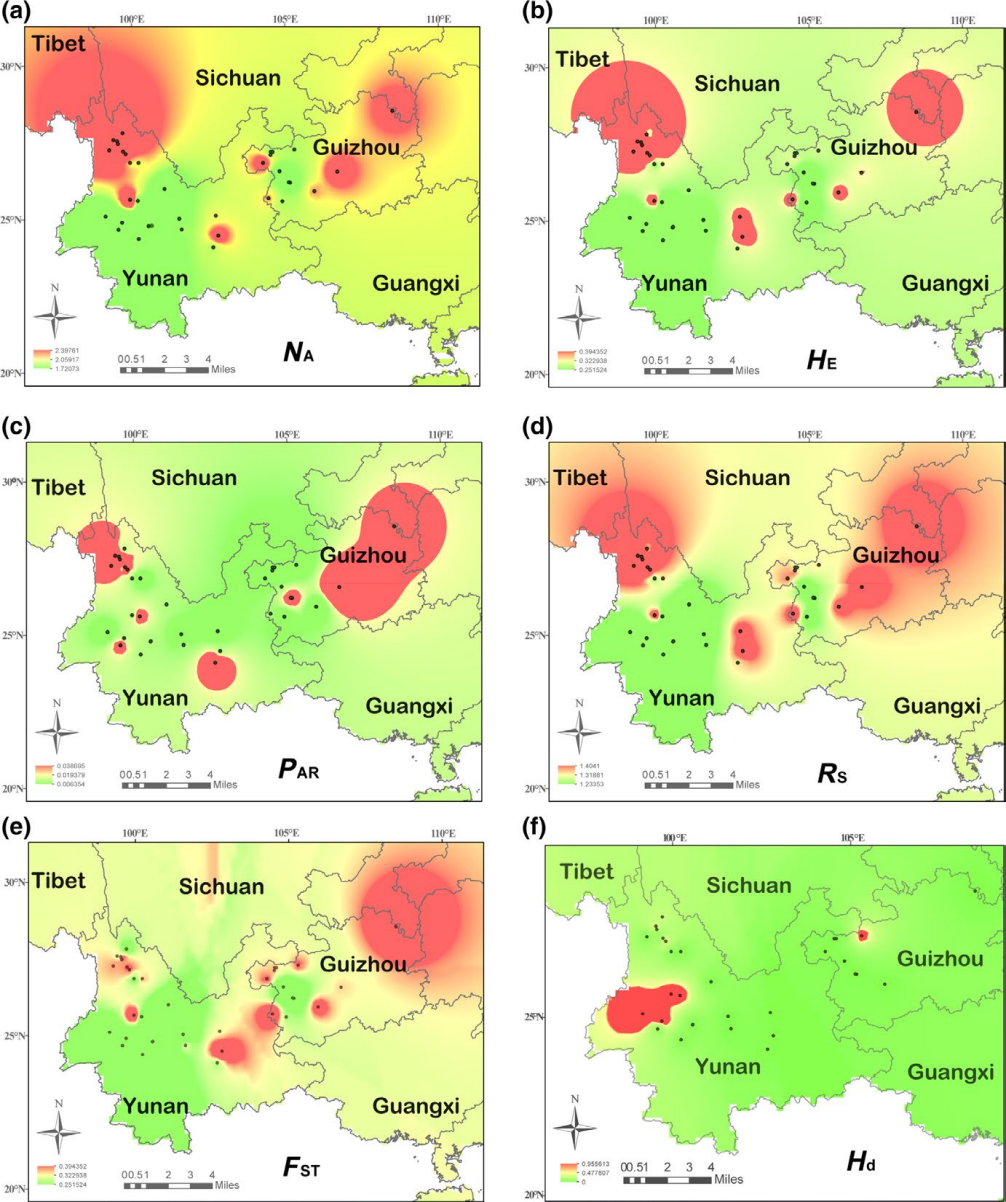
Inverse distance weighted (IDW) interpolation of the number of alleles (*N*_A_), a), expected heterozygosity (*H*_E)_, b), private allelic richness (*P*_AR_), c), allelic richness (*R*_S_), d), genetic differentiation (*F*_ST_), e), and haplotype diversity (*H*_d_), f) calculated for 36 iron walnut populations (black dots) in China based on 20 EST‐SSR markers. Details concerning the locations of populations and their sample sizes can be inferred from a material table provided at Dryad under https://doi.org/10.5061/dryad.h70rxwddv

For each population of *J. sigillata*, the number of alleles (*N*_A_) and the number of effective alleles (*N*e) varied from 1.77 to 3.18 and from 1.44 to 2.07, respectively. The observed heterozygosity (*H*_O_) ranged from 0.16 to 0.41. Allelic richness (*R*_S_) ranged from 1.23 to 1.41 with average of 1.31. Screening all 765 *J. sigillata* individuals at the 22 EST‐SSR loci identified a total of 99 alleles, the number of alleles (*N*_A_) per locus for the 22EST‐SSR ranged from 2 to 12 with a mean of 4.85 per locus (Table S2). The gene diversity within populations (*H*s) ranged from 0.018 to 0.596 (Table S2). The IDW analysis showed that high values of *H*_E_, *R*_S_, *N*_A_, and *P*_AR_ were observed in populations from the northwestern geographic area of the species distribution, close to Hengduan Mountains, and in a few populations of eastern Yunnan province and Guizhou province (Figure 2). The allelic richness (*R*_S_) ranged from 1.23 (MD and ST populations) to 1.41 (TZ). High values of allelic richness (*R*_S_) were observed in populations from the northwestern and northeastern margins of the species distribution and from some populations in eastern Yunnan. Private allelic richness (*P*_AR_) was highest in population (JL) from eastern Yunnan province (*P*_AR_ = 0.04). High values of the number of alleles (*N*_A_) were observed in areas near the Hengduan Mountains, where there were also some populations with high values of expected heterozygosity. High values of genetic differentiation (*F*_ST_) were observed in areas near the Wuling Mountains and Da Luo Mountains in Guizhou province.

### 3.3 Population structure based on EST‐SSR data

**Figure 3:**
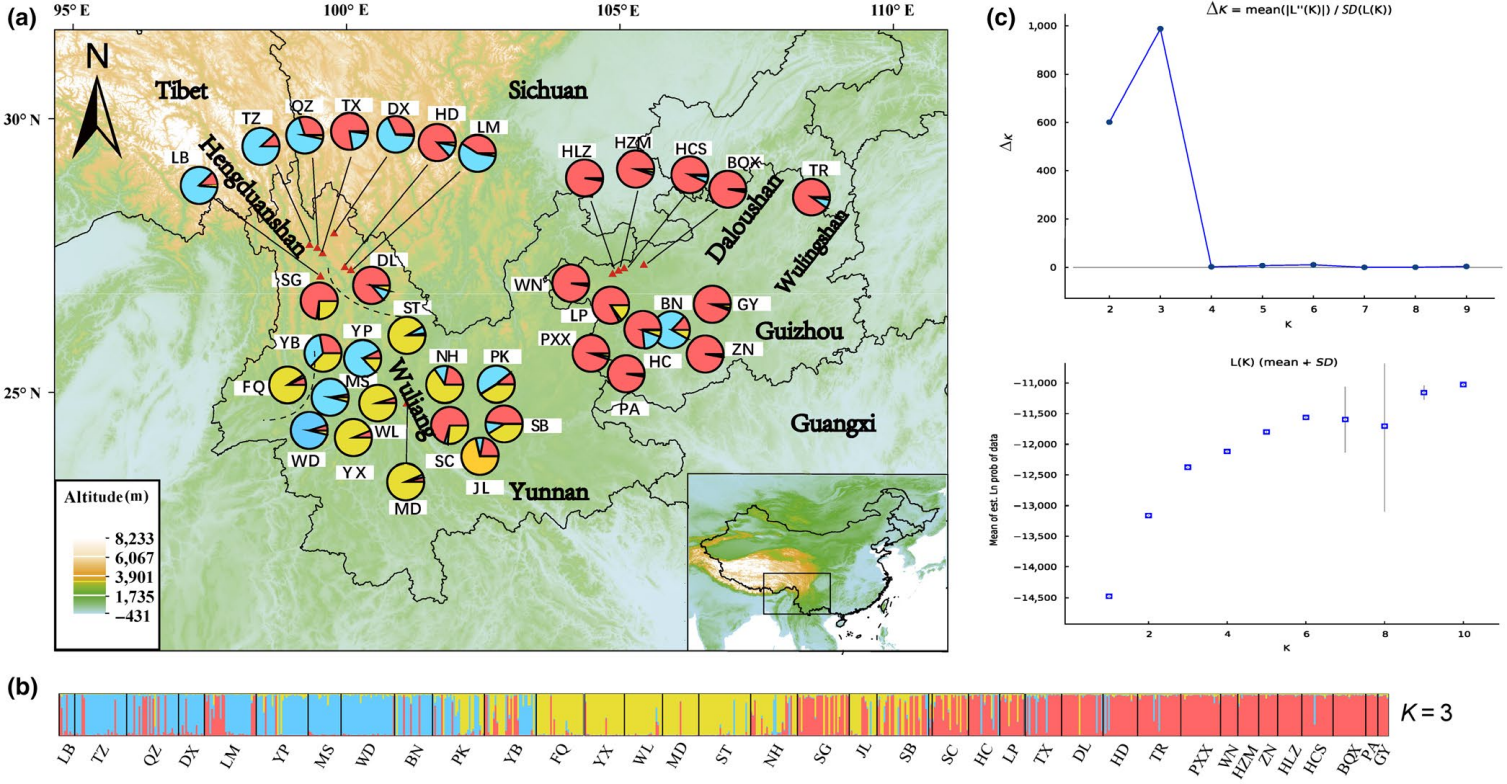
(a) Locations of the 36 populations of *Juglans sigillata* at 12 neutral loci and their color‐coded grouping at the most likely *K* = 3. (b) Histogram of the STRUCTURE analysis for the model with *K* = 3 (showing the highest Δ*K*). Each color corresponds to a suggested cluster, and a vertical bar represents a single individual. Population codes are indicated below. (c) Distribution of delta *K* for *K* = 3 to 10 to determine the true number of populations (*K*) as described in Evanno et al. (2005). Mean log likelihood of the data at varying estimates of *K*

Among the 22 EST‐SSR loci we examined, eight showed evidence of positive selection with 95% confidence intervals (CI) based on *F*_ST_ outliers (Figure S1). Applying Bayesian analysis of genetic structure to the trees from all 36 iron walnut populations using only 12 neutral loci, the most likely number of populations was *K* = 3 (Figure 3c). For *K* = 3, nine populations from northern Yunnan province and southern Yunnan province and only one population (BN) from Guizhou province clustered into population I (Figure 3; blue color); samples from eight demes, situated in the middle of Yunnan province clustered into population II (Figure 3; yellow color); samples from eighteen demes clustered into populations III (Figure 3; red color), including six demes from Yunnan province, and twelve populations from Guizhou province (Figure 3). However, the iron walnut samples separated into two subpopulations based on all EST‐SSR markers (cluster A and cluster B in Figure S2). The second highest peak for Δ*K* was 3. Applying Bayesian analysis of genetic structure, all 36 sampled *J. sigillata* locations plus 12 *J. regia* sites using only the 12 neutral loci, and the most likely number of populations was *K* = 3 (Figure S3).

### 3.4 Genetic differentiation, gene flow, and genetic barriers based on cpDNA and EST‐SSR

The genetic differentiation among iron walnut populations (*G*_ST_) was 0.194, and *N*_ST_ was 0.222 (*N*_st_ − *G*_st_ = 0.028; *p* = .202), indicating the nonexistence of a phylogeographic pattern. Neutrality tests (Tajima's *D* = 0.113, *p* > .5; Fu's *F*s = 0.903, *p* > .5) on cpDNA did not detect evidence of recent range expansion. The multimodal mismatch distributions indicated that the *J. sigillata* populations had a stable range (Figure 1c).

The AMOVA on cpDNA and EST‐SSR data provided evidence for a relatively high level of genetic differentiation among sampled sites; a considerable percentage (24.72% and 17.42%, respectively) of the genetic variation was attributed to differences among sampled location (Table 1). Population differentiation was significant at 20 loci (*p* < .05), with the average *F*_ST_ equal to 0.262 (range from 0.088 to 0.800). The pairwise *F*_ST_ values ranged from 0.021 (TR vs. HZM) to 0.265 (MD vs. HC). Population MD showed the highest mean pairwise *F*_ST_ with other populations (0.166). A Mantel test indicated no significant correlation between the genetic distance and geographic distance among populations based on nSSR data (*r* = .011, *p* = .400).

**Table 1:** Analysis of molecular variance (AMOVA)

Source of variation	*df*	SS	VC	PV (%)	Fixation index
cpDNA					
Among population	30	105.70	0.3896	24.72	*F*_ST_ = 0.247**
Within population	155	183.83	1.1860	75.28	
Total	185	289.53	1.5756		
Microsatellite					
Among populations	35	235.714	0.14	17.42	*F*_ST_ = 0.17**
Within populations	1,492	1,012.663	0.68	82.58	
Total	1,527	1,248.377	0.82		
All samples^a^					
Among species	1	19.63	0.016	1.98	*F*_ST_ = 0.18**
Among populations within species	34	216.086	0.135	16.23	*F*_SC_ = 0.17**
Within populations	1,492	1,012.663	0.679	81.79	*F*_CT_ = 0.02*
Total	1,527	1,248.377	0.82986		

Abbreviations: *df*, degrees of freedom; *F*_CT_, differentiation among groups within three species; *F*_SC_, differentiation among populations within species; *F*_ST_, differentiation among populations within three species; PV, percentage of variation; SS, sum of squares; VC, variance components.

a Indicate that all populations combined with *J. regia* and *J. sigillata*.

* *p* < .05, ^**^ *p* < .01, 10,000 permutations.

Based the three populations of iron walnut (*K* = 3; Figure 3), the gene flow analysis showed that almost all the historical gene flow of the related pairs was symmetrical; between populations II and III, there was slightly asymmetric gene flow historically. The gene flow (2*Nm*) from population II to population III was 5.64 (95% CI: 0.025–0.995; Table S3), whereas in the opposite direction, it was predicted to be 2.34 (95% CI: 0.025–0.995; Table S3). We located three statistically significant barriers to gene flow when all iron walnut populations were included using Monmonier's maximum difference algorithm (Figure S5).

### 3.5 Divergence times and historical biogeographical inference

**Figure 4:**
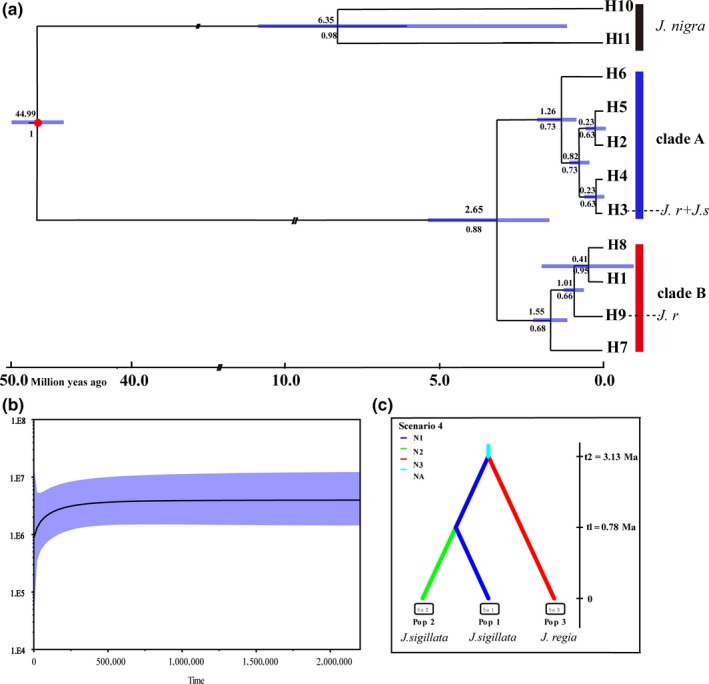
(a) BEAST‐derived chronograms of 11 haplotypes of *Juglans sigillata*, *Juglans regia*, and *Juglans nigra* based on five chloroplast DNA (cpDNA) fragments. (b) Bayesian skyline plot derived from 186 sequences of *Juglans sigillata*. The thick solid line represents the median estimate, and the light blue area corresponds to the 95% highest posterior density (HPD). The divergence times estimated using a relaxed molecular clock model with fossil data (red dots). (c) The three scenarios tested in DIYABC. In these scenarios, *t*# represents timescale in terms of the number of generations and *N*# represents the effective population size during the time period (e.g., 0–*t*1, *t*1–t2). The summary shows scenario 4 is the most likely scenario in DIYABC

The BEAST‐derived cpDNA chronogram indicated a Mid‐Eocene split between *J. sigillata* (sect. *Dioscaryon*) and the out‐group (*J. nigra*, sect. *Rhysocaryon*) about 44.99 Ma (Figure 4a). The calibration note was based the fossil data within *Juglans* (divergence of black walnut, *J. nigra*, 38 ~ 45 Ma) (Bai et al., 2016). All haplotypes of *J. sigillata* clustered into two clades (clade A and clade B), which diverged from each other during the Pliocene–Pleistocene (2.65 Ma, Figure 4a). A point estimate for the coalescent time for clade A was dated to 2.65 Ma (Figure 4a). The divergence time of the early‐diverging clade B was estimated at 1.55 Ma and that of the H1 and H8 lineage at 0.41 Ma (Figure 4a). All major divergence events within each of the two major lineages/clusters were assigned to the late Tertiary and the early Quaternary (Figure 4). Based on these three metapopulations (populations 1, 2, and 3; Pop 1: *J. sigillata*; Pop 2: *J. sigillata*; Pop 3:12 populations of *J. regia*), we evaluated 13 scenarios related to the phylogeny and demography of *J. sigillata* and the relationship of *J. sigillata* and *J. regia* in China (Figure 4c; Figure S3; Table S4). DIYABC unambiguously indicated support for scenario 4 (0.3517, 95% CI: 0.3262–0.3772), the 95% confidence intervals of this model did not overlap with the 12 other scenarios (Figure S4). Scenario 4 posited population 3 (*J. regia*) at t2 (3.13 Million years ago, during the Pliocene) and a more recent separation of two subpopulations of *J. sigillata* at t1 (0.78 Million years ago, Pleistocene, in the Quaternary) (Figure 4).

### 3.6 Iron walnut distribution during the Late Quaternary

**Figure 5:**
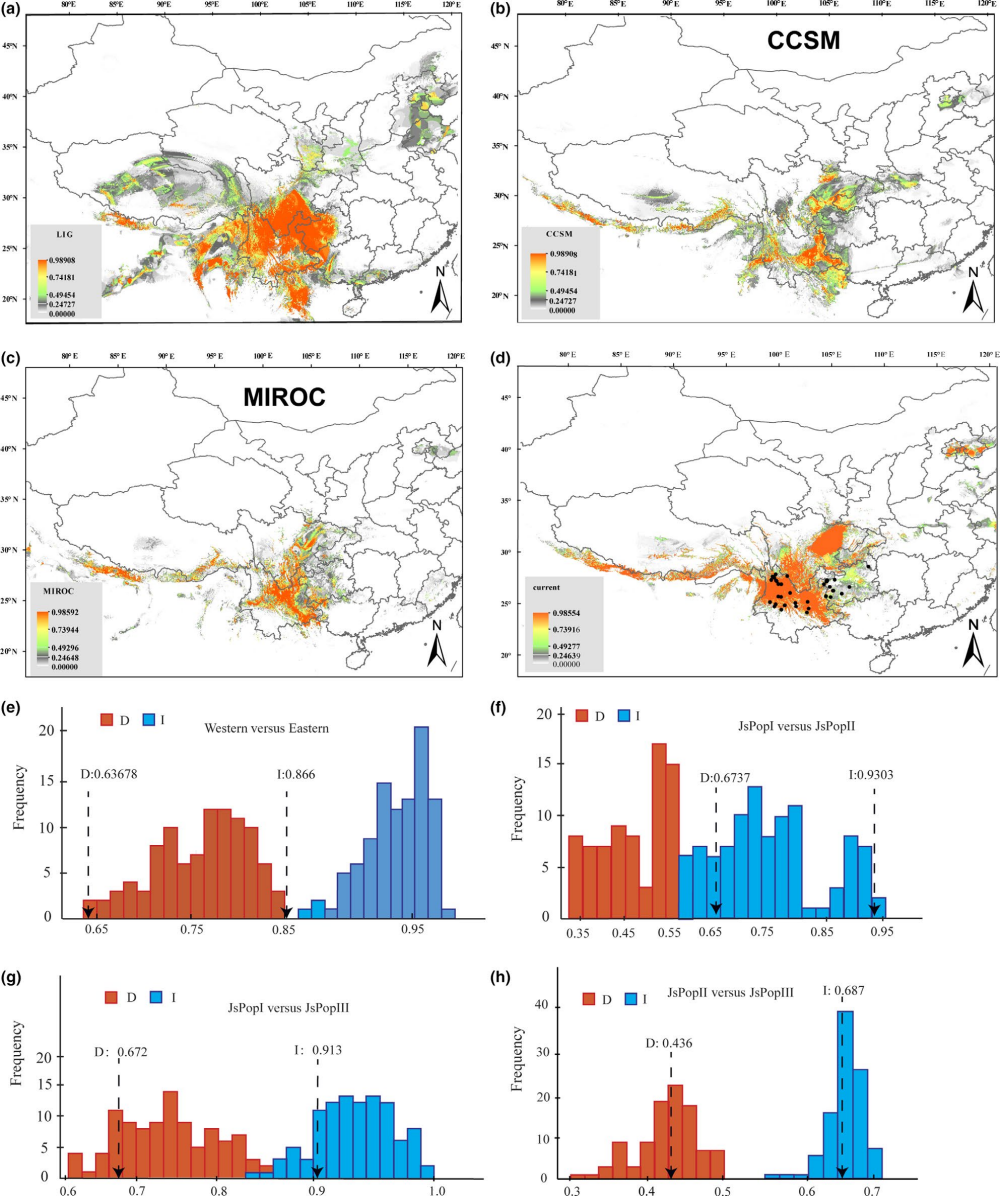
Results of ecological niche models of *Juglans sigillata*. (a) Average projection of the model to the last interglacial (*c*. 120–140 kyr BP). (b and c) Average projections of the model to the last glacial maximum [*c*. 21 kyr before present (BP)] using climatic variables under the Community Climate System Model (CCSM) (b) and Model for Interdisciplinary Research on Climate (MIROC) and (c) general circulation model simulations. (d) Predicted distribution probability for current climatic conditions. The map was produced using ArcGIS. (e) Niche identity tests of western population *J. sigillata* and eastern population of *J. sigillata* (Figure 1). (f) Niche identity tests for population I and population II of *J. sigillata* (Figure 3). (g) Niche identity tests for population I and population III of *J. sigillata* (Figure 3). (h) Niche identity tests for population II and population III of *J. sigillata* (Figure 3). Null distributions are shown by dotted blue bars for *D* and solid red bars for *I*. The *x*‐axis indicates values of *I* and *D*, and *y*‐axis indicates number of randomizations. Two arrows indicate values in actual MAXENT runs, respectively

The Maxent model of *J. sigillata* was supported by a high predictive power, with the AUC = 0.996 ± 0.0002 (mean ± *SD*). The projection of the model fit to current climate conditions indicated suitable habitats in southwest China (current) between about 22°N and 32.5°N and most of Yunnan province (Figure 5d), plus a few additional areas on the coast of the Bohai Sea. With 0.75 chosen as the threshold suitability, *J. sigillata* was predicted to have occurred (during the Quaternary) in a slightly more southern range (around 15°N–30°N) during the LIG (Figure 5a); during LGM, suitable habitat was predicted between 22.5°N and 32°N (Figure 5b,c), which included much of what is now Yunnan province and a small part of Sichuan and Guizhou provinces. The predicted distribution of *J. sigillata* during LGM was markedly reduced compared with the LIG, probably due to colder climates predicted under both the CCSM and MIROC models during LGM. We learned that temperature and precipitation were the most important factors driving the distribution of *J. sigillata* (Figure 5). Based on the results of the niche identity test, the observed niche overlap values for *I* and *D* were significant for the western population and eastern population of *J. sigillata* (Figure 5; Figure 1 shows the two populations based on chloroplast sequence data), indicating that the niches of two subpopulations were different. However, the ranges of the three predicted *J. sigillata* populations (populations I, II, and III, Figure 3; *K* = 3 in STRUCTURE analysis) (Figure 5f,g,h), and only niche overlap values of population I versus population III for *I* and *D* were significantly different (Figure 5g). Inspection of the spatial overlap between Ecological Niche Models (ENMs) revealed that factors other than those described in the ENM may maintain parapatry for *J. sigillata* genetic subpopulations (Figure 5).

## 4 DISCUSSION

### 4.1 The relationships of *J. regia* and *J. sigillata*

Southwestern China is hot spot of biodiversity and endemism for temperate plant species (López‐Pujol et al., 2011) and believed to have been the location of important glacial refugia for a number of Tertiary relict trees (Qiu, Fu, & Comes, 2011). *Juglans sigillata* is indigenous to China, distributed mainly in SW China sympatric with *J. regia* (Wang et al., 2014). *Juglans regia* and *J. sigillata* were designated as the sole members of section *Juglans* (Gunn et al., 2010).

Studies of gene flow and introgression have concluded *J. regia* and *J. sigillata* are particularly closely related, and some have questioned whether they are distinct (Wang et al., 2015, 2008). Aradhya et al. (2007) considered *J. sigillata* distinct based upon ITS, RFLP, and cpDNA sequence data and morphology. We found that the *J. sigillata* samples appeared completely embedded within *J. regia* based on a phylogeny analysis using whole genome data (Zhao et al., 2018). Our data provide the evidence that *J. sigillata* is a subspecies or, perhaps, a landrace of *J. regia* (Figure 4; Figure S3).

Shared chloroplast haplotypes among closely related populations can be explained by incomplete lineage sorting, intraspecific gene exchanges, or a shared most recent common ancestor (Petit, Bodénès, Ducousso, Roussel, & Kremer, 2003; Yang et al., 2016). In this study, we used nuclear microsatellite and cpDNA markers and ecological niche modeling to reconstruct the phytogeographic history of *J. sigillata* and identify forces that most influenced the species' genetic structure.

### 4.2 Patterns of genetic diversity and genetic differentiation

We found the centers of genetic diversity of *J. sigillata* were located at Hengduan Mountains, Dalou Mountains, and small region of Yunnan province based on IDW analysis (Figure 2). The chloroplast haplotype H3 (which is also found in *J. regia*) was found in trees across the entire range of *J. sigillata* [with high SSR genetic diversity that could be gene flow from *J. regia* (Figure 1). The diversity of chloroplast haplotypes was also high in populations from Hengduan Mountains, Dalou Mountains, Wuling Mountains, and a small region of Yunnan province (Figure 2). These results indicated that hybridization and gene introgression from *J. regia* may affect the genetic diversity of *J. sigillata* in these areas, resulting in populations with high diversity (Figures 1 and 2). From our point of view, it is essential to evaluate the geographical patterns of genetic diversity and identify the populations and areas that show high values of genetic diversity and divergence for conservation and germplasm collection. Comparing the spatial representation of *H*_E_, *R*_S_, *N*_A_, *P*_AR_, and *F*_ST_, high values of these genetic diversity indexes were observed in the Hengduan Mountainous Region, the Wuliang mountains, and the northeastern Yunnan–Guizhou plateau, near Tongren. These may be regarded as centers of genetic diversity (Figure 2), and possibly refugia during LGM.

The most important forces affecting the spatial genetic structure of *J. sigillata* are those related to dispersal and interactions with humans. In natural walnut stands, pollen‐mediated gene flow probably rarely disperses alleles across distances >300 m (Pollegioni et al., 2015). Walnut seeds are large and heavy, so their dispersal by granivores (mostly squirrels) likely takes place over even more limited distances. As an economically important tree species, walnut plants were inevitably disturbed by human activities such as deforestation and selection (Lee & Lee, 1997), particularly over the past 100 years. Human movement of germplasm (seeds as well as clones) can mix haplotypes much faster than chloroplast capture in natural populations. Human exploitation of a wild resource often results in a decline in effective population size (*N*e), loss of genetic diversity, population fragmentation, and local extirpation (Lefèvre, 2004; Wang et al., 2008). Tree species that grow in close association with humans are subject to unique evolutionary and ecological processes. For instance, artificial selection pressures lead to morphological changes in cultivated populations, dispersal by humans expands the natural range of species, and range expansion can lead to sympatry and hybridization with otherwise allopatric congeneric species like *J. cathayensis*. Humans likely contributed to both local and long‐range dispersal of *J. sigillata* seeds through trade (Gunn et al., 2010). Aside from human dispersal, differences in the frequency of self‐pollination, geographic isolation, local adaptation, phenological differences, and stochastic events including disease or pest outbreaks are all likely to have affected the spatial genetic structure of iron walnut.

Generally, *F*_ST_ estimates for cytoplasmic markers are higher than for nuclear markers (Chen, Lu, Zhu, Tamaki, & Qiu, 2017; Petit et al., 2005). This was not true, however, for *J. sigillata*. Iron walnut expressed comparatively low levels of population differentiation at maternally inherited cpDNA markers (*G*_ST_ = 0.194). The two most likely causes for this result are first, recent sporophytic gene exchange between populations that had, until recently, been isolated, or second, recent fragmentation of a large population with recurrent sporophytic gene flow. If *J. sigillata* populations had a history of long‐term isolation, then we might expect each population would have independently experienced genetic drift, resulting in a high frequency of rare haplotypes in each population (Figure 1). The second hypothesis is that populations were recently separated, so there has not been enough time for the effects of genetic drift to appear in each small, fragmented population. If the second hypothesis is correct, we would expect that most populations would share common haplotypes (Printzen, Ekman, & Tønsberg, 2003). In support of the second hypothesis, except for two haplotypes (H2 and H5) specific to population QZ and LM, the remaining haplotypes were widely shared among populations, especially H3, which was found in all populations (Figure 1a). Therefore, the hypothesis of recent fragmentation of a large population with recurrent gene flow happened historically is more probable. This hypothesis is corroborated by ABC and BEAST analysis (Figure 4). What's more, the overall *F*_ST_ value of 0.266 from nuclear microsatellite data is relatively high as compared with other wind‐pollinated temperate trees (Lind & Gailing, 2013; Rusanen, Vakkari, & Blom, 2003). Maybe the gene flow among populations now is mostly humans moving nuts. Besides, there was no significant difference in the frequency of the private alleles in all populations that might also indicate recent fragmentation of a metapopulation. Generally, *J. sigillata* grows on mountain slopes or in valleys. Southwest China is characterized by strong local variation in climate due to complex topography. Increases in fragmentation result in smaller effective population sizes, lower gene flow among populations and even to a higher *F*_ST_ (Printzen et al., 2003).

### 4.3 Population structure, barrier, and gene flow

Considering the structural and geomorphological complexity of Yunnan–Guizhou plateau regions, it is likely that geographical barriers have interfered with the gene flow between iron walnut populations. *F*_ST_ > *G*_ST,_ it manifest that the most likely cause of this situation in the natural state is the geographical isolation caused by the geographical environment. Geographic features explain the genetic structure obtained from STRUCTURE analyses of EST‐SSRs in *J. sigillata*. When *K* = 3, there was evidence for some gene flow from populations in Yunnan province and populations in Guizhou province (Figure 3; Table S3). According to the previous studies, natural gene flow across large distances in SW China was unlikely, perhaps indicating the importance of human movement of *J. sigillata* seeds (Wang et al., 2015). An important result from our investigation is that gene flow rate between pairs of populations does not correlate with their geographical distances (*r* = .011, *p* = .400), providing a potential explanation for why a Mantel Test comparing the matrix of genetic and geographic distance was not significant (Bai et al., 2016).

Iron walnut is a wind‐pollinated tree, the seeds or pollen dispersed by winds, born at 1,300–3,300 m above sea level on hillsides or valleys. In Yunnan–Guizhou plateau region, especially Yunnan province, most of the areas are characterized with highly dense mountain streams and lush vegetation. It maybe has served as effective barriers to seed and pollen dispersal and reduced the potential for long‐distance seed dispersal. The genetic barrier analysis revealed three mains statistically barrier exist among populations, including the barrier separated population DL from four adjacent populations (SG, YB, YP, and ST). The other two barriers also effect gene flow between populations of *J. sigillata* (Figure S5). This may indicate that local populations suffer from a deficit in outcross pollen, and inbreeding depression due to selfing or mating between close relatives (Qiu, Luo, Comes, Ouyang, & Fu, 2007). Values of *F*_IS_ per population ranged from −0.29 to 0.28, with an average of 0.05, indicating an overall slightly excess of homozygotes. In other words, a selfing breeding system might exist in *J. sigillata* that is known to be self‐compatible. IBD was significant for *J. sigillata* in SW China (Figure S5), so geographical distance likely restricted gene flow and increased genetic differentiation (reflected in high *F*_ST_ values).

### 4.4 Population history and range dynamics

Phylogenetic analyses using both Bayesian and parsimony methods partitioned our samples into two distinct cpDNA clades (Figure 4a). Differentiation time of *J. regia* and *J*. *sigillata* was 3.13 Ma, and it showed two species separated in the Pliocene–Pleistocene. Using a Bayesian dating method, we estimate the divergence times between clade A and clade B to be 2.65 Ma (Figure 4a). Our molecular dating by cpDNA and SSR data both fell into the early Pliocene–Pleistocene. The uplift of the Yunnan–Guizhou plateau in SW China likely occurred in the late Miocene–Pliocene (Clark et al., 2004; Favre et al., 2015), a period of global cooling and intensification of Asian monsoons. Habitat fragmentation caused by the Yunnan–Guizhou plateau uplift during Miocene–Pliocene may have fostered intraspecfic divergence in this region (Feng et al., 2016; He & Chen, 2006).

The current geographical distribution of *J. sigillata* appears to reflect three scattered refugia in the Yunnan–Guizhou plateau, Hengduan mountain, areas near the Wuliang mountains, and a refugium somewhere in the northeastern portion of the species' distribution (near Dalou mountain and Wuling mountain). These putative refugia have been recognized as centers of plant diversity. However, the refuge of the northeastern species distribution has not appeared in the SDM model, and this might be because populations along this potential route may have gone extinct, but it will occur again in the simulation of future (Figure 5e). Our SDM analyses also suggested that *J*. *sigillata* had experienced ranges shrinkage, retreated to the southern region about 25°N from LIG to LGM within refugia, which was similar to *Juglans regia* (Feng et al., 2018). Then, the species underwent a slight northeastward expansion, but the effective population size did not exceed LGM. This phenomenon is the same as the result of the BSP (Figure 4c); overall, the effective population experienced a reduction at 0.25 Ma in the process. In general, the most reason is that increasingly colder and arid environment has occurred in LGM; afterward, climate is getting warmer.

## 5 CONCLUSIONS

In short, we integrated molecular phylogeography, SDMs, and phylogenetic approaches to understand intraspecific divergence, evolutionary history, and range dynamics of *J*. *sigillata*. Using coalescent‐based methods and ABC analysis, we determined the divergence of this subspecies to have occurred about 0.78 Million years ago, during the Pleistocene. It may have been markedly affected by geological and climatic changes since Miocene linked to the uplift of the Yunnan–Guizhou plateau. It may even be a split from a common walnut (*J. regia*), which requires further research. The genetic analyses and SDMs indicated different phylogeographical patterns follow the species routes during the interglacial–glacial climatic oscillations of the Quaternary. Our study provides an additional case to help understand the origin and spread routes of the extraordinary biodiversity of the SW China.

## CONFLICT OF INTEREST

None declared.

## AUTHOR CONTRIBUTIONS

PZ and YWS involved in conceptualization. PZ, YWS, NH, and KW curated the data. PZ, YWS, NH, CZ, and XY performed formal analysis. PZ funded the acquisition. PZ, YWS, NH, CZ, and XY investigated the study. PZ, YWS, NH, CZ, and XY wrote the original draft. PZ, YWS, NH, and KW revised the manuscript.

## Data Availability

All newly obtained cpDNA sequences (MH606007–MH606019) and were uploaded to GenBank. Information of samples and genotype data can be inferred from a material table provided at Dryad under https://doi.org/10.5061/dryad.h70rxwddv.
