# Supplementary material for: Population genetic structure and adaptive differentiation of iron walnut Juglans regia subsp. sigillata in southwestern China
Source: Ecol Evol. 2019 Nov 21;9(24):14154–66. doi: 10.1002/ece3.5850 (PMC6953554; doi:10.1002/ece3.5850)
Supplement: Supplementary file 1 [file ECE3-9-14154-s001.docx]

**Supporting information for manuscript entitled:** **P****opulation genetic structure and adaptive differentiation of iron walnut *Juglans regia* subsp. *sigillata* in southwestern China**

**Yi-Wei Sun^1†^, Na Hou^2†^, Keith Woeste^3^, Chuchu Zhang^1^, Ming Yue^1,4^, Xiao-Ying Yuan^1^, Peng Zhao^1*^**

^1^Key Laboratory of Resource Biology and Biotechnology in Western China, Ministry of Education, College of Life Sciences, Northwest University, Xi’an, Shaanxi 710069, China

^2^Guizhou Academy of Forestry, Guiyang, China

^3^USDA Forest Service Hardwood Tree Improvement and Regeneration Center (HTIRC), Department of Forestry and Natural Resources, Purdue University, 715 West State Street, West Lafayette, Indiana, 47907, USA

^4^ Xi’an Botanical Garden of Shaanxi Province, Xi’an, Shaanxi 710061, China;

† These authors contributed equally to this work

***Correspondence author:** Peng Zhao

Email address: [pengzhao@nwu.edu.cn](mailto:pengzhao@nwu.edu.cn)

Tel: +86-029-88302411

Address: 229 Taibaibei Road, Xi’an, Shaanxi, China 710069


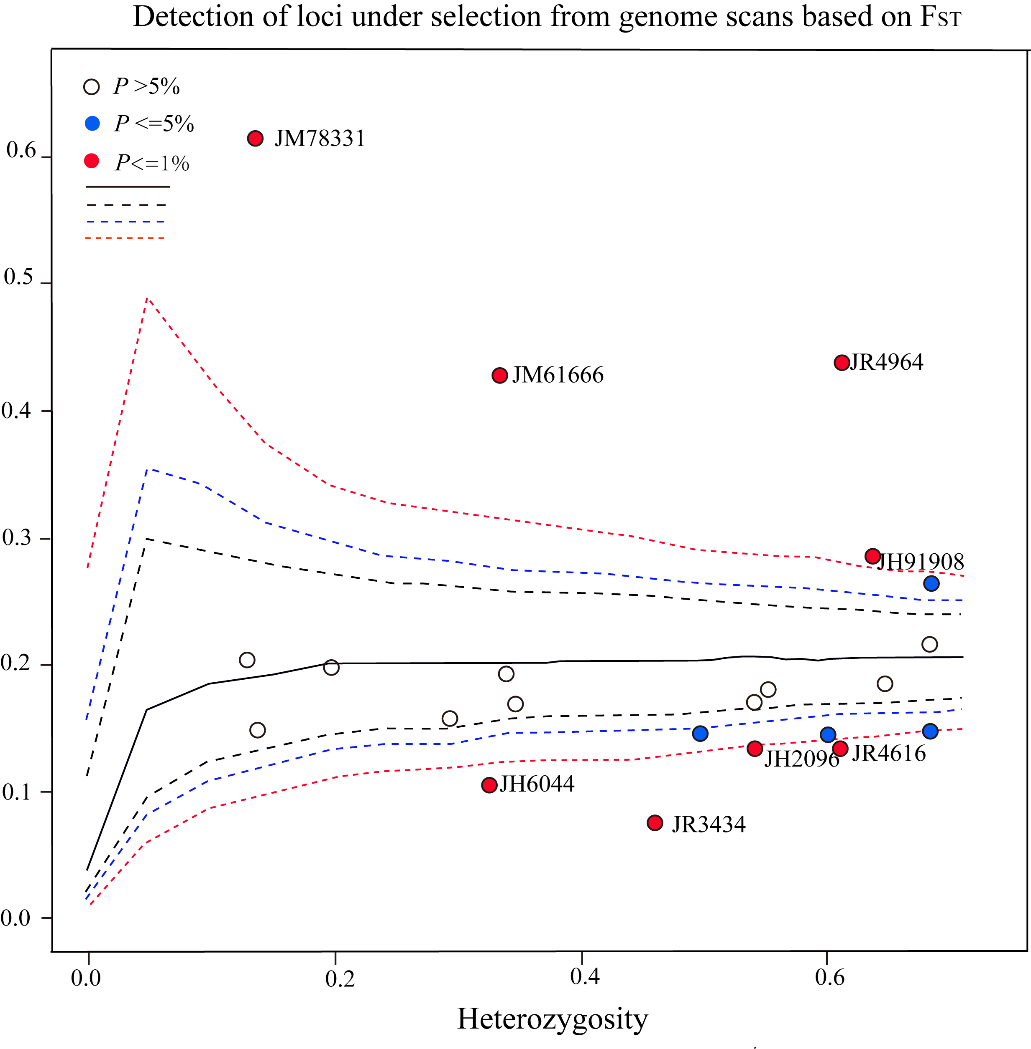


**Figure S1** Detection of loci under selection from genome scans based on F_ST_ testing by software Arlequin 3.5.


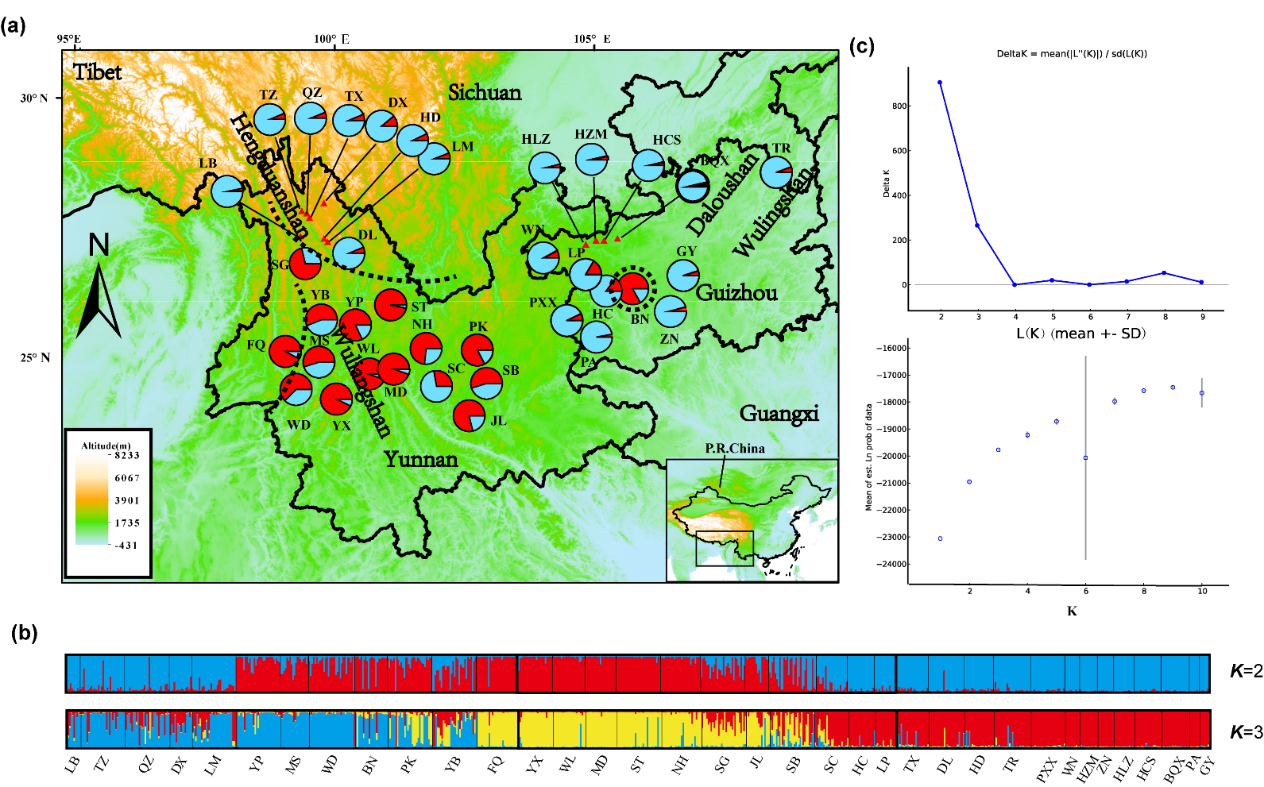


**Figure S2** (a) Locations of the 36 populations of *Juglans sigillata* based on all EST-SSR loci and their color-coded grouping at the most likely *K* = 2. (b) Histogram of the STRUCTURE analysis for the model with *K* = 2 and *K* = 3. Each color corresponds to a suggested cluster, and a vertical bar represents a single individual. Population codes are indicated below. (c) Distribution of delta *K* for *K* = 2 to 10 to determine the true number of populations (*K*), and mean log likelihood of the data at varying estimates of *K* (described in Evanno (2005)*.*


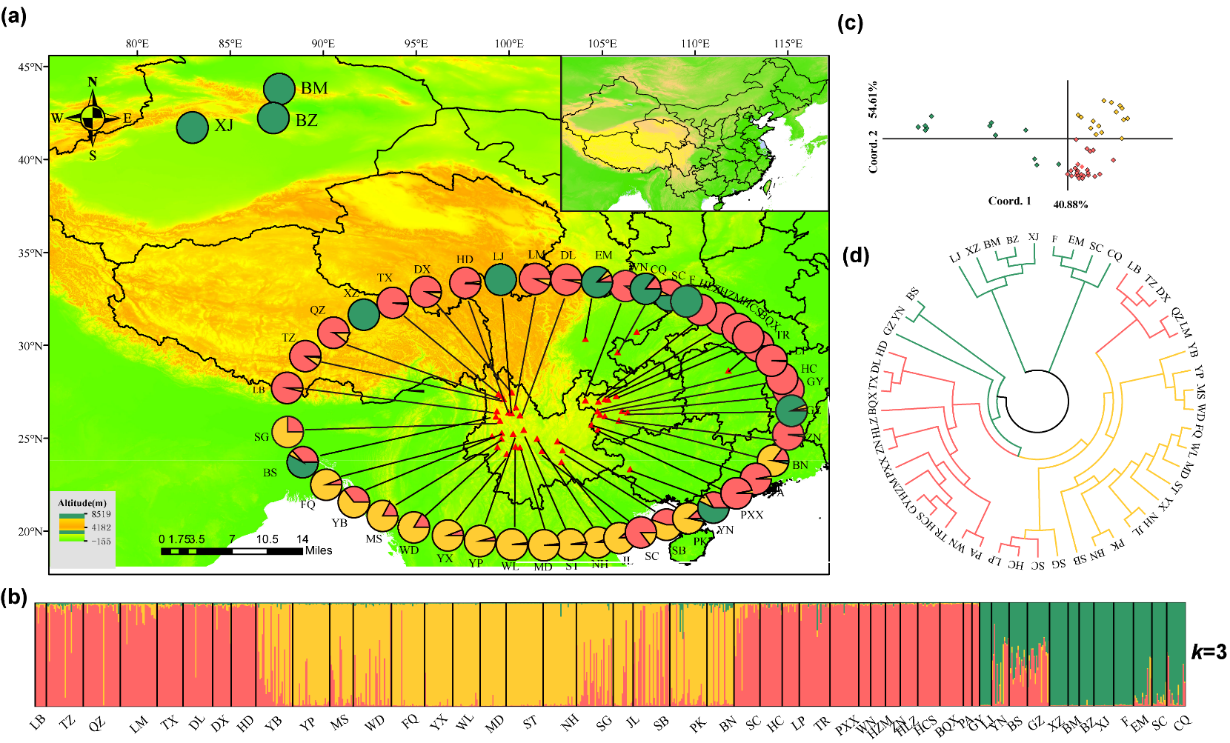


**Figure S3** (a) Locations of the 36 populations of *Juglans sigillata* and the 12 populations of *Juglans regia* based on all EST-SSR loci and their color-coded grouping at the most likely *K* = 3. (b) Genetic clusters histogram of two walnut population using by STRUCTURE analysis for the model with *K* = 3. Each color corresponds to a suggested cluster, and a vertical bar represents a single individual. Population codes are indicated below*.* (c) Principal coordinate analyses (*PCoA*). (d) Neighbor Joining phylogenetic analysis.


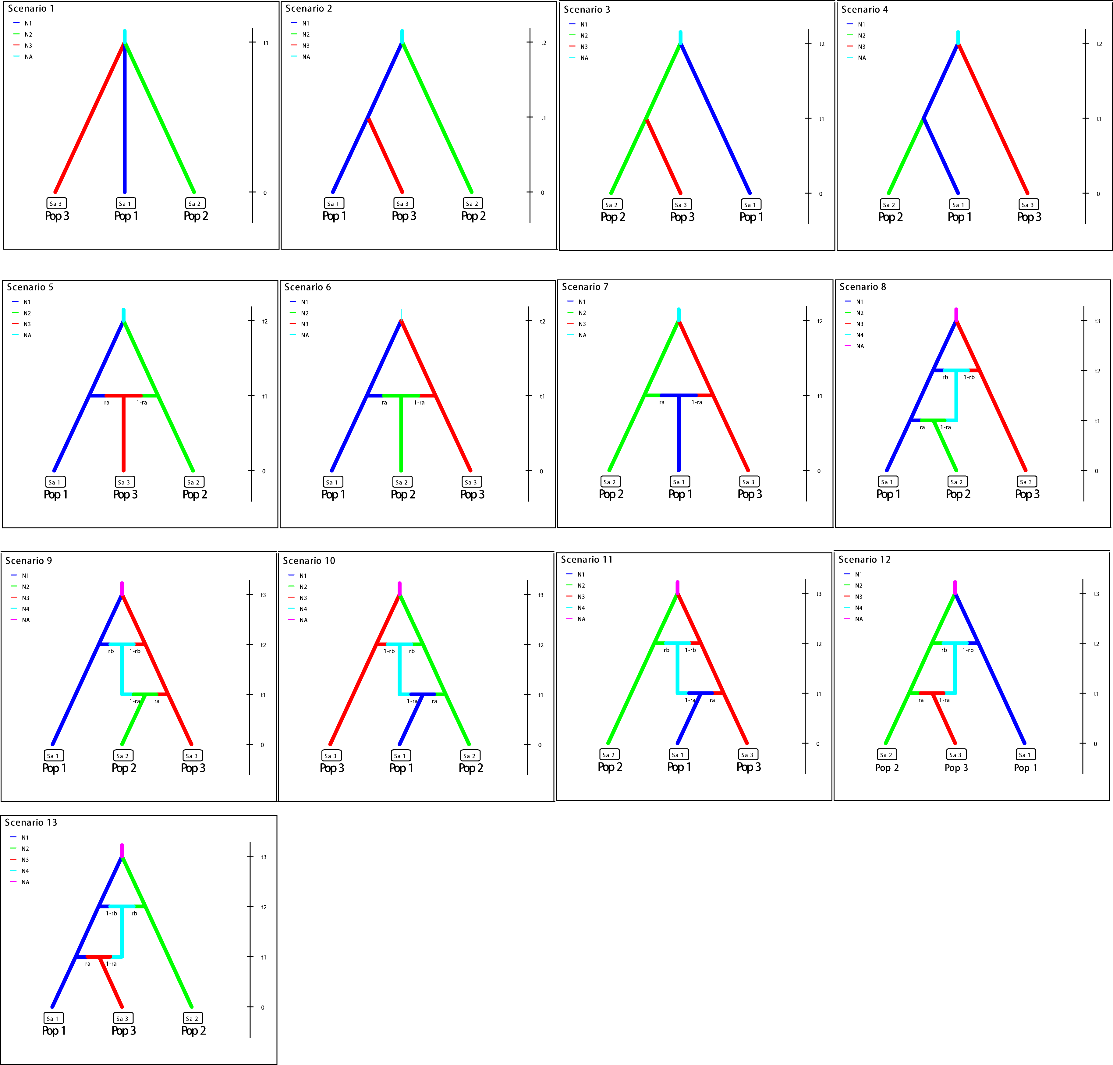


**Figure S4** Thirteen scenarios of *J. sigillata* and *J. regia* in DIYABC.


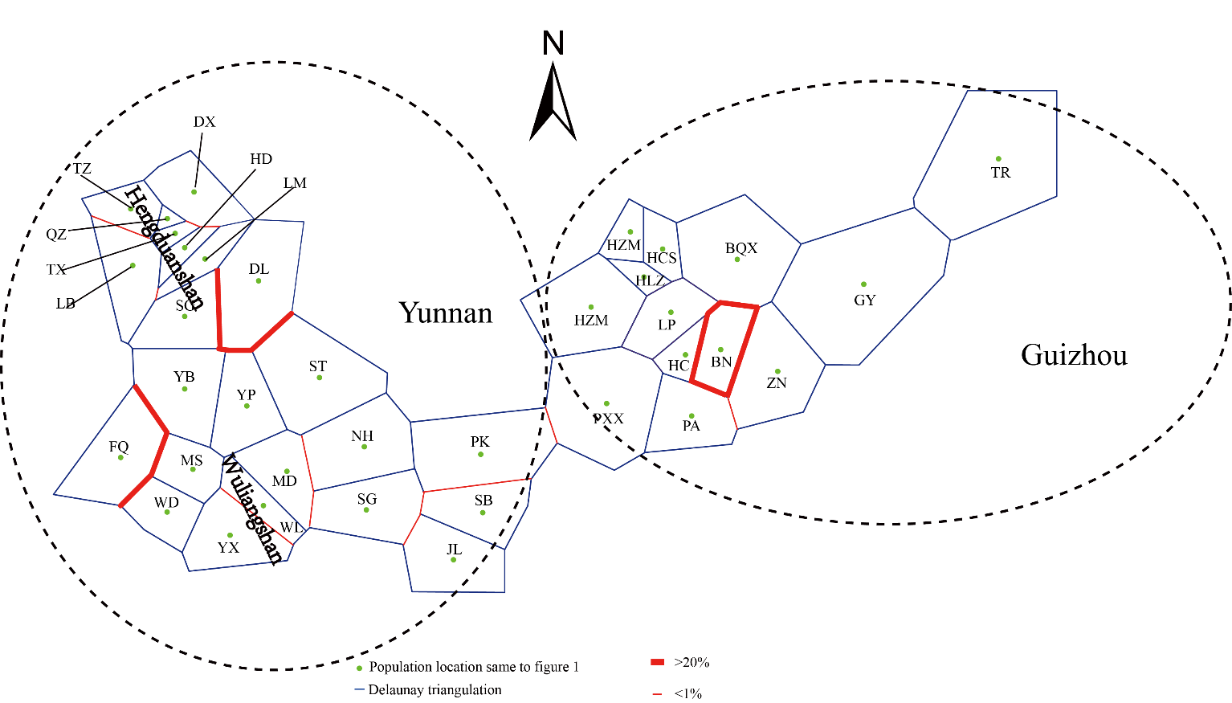


**Figure S5** Results of the BARRIER analysis based on microsatellite data, showing the spatial separation of *J. sigillata* populations. Geographic location of the genetic barrier, indicated by red line, as suggested in the BARRIER analysis.
