# Supplementary material for: Population genetic structure and adaptive differentiation of iron walnut Juglans regia subsp. sigillata in southwestern China
Source: Ecol Evol. 2019 Nov 21;9(24):14154–66. doi: 10.1002/ece3.5850 (PMC6953554; doi:10.1002/ece3.5850)
Supplement: Supplementary file 2 [file ECE3-9-14154-s002.docx]

**Supporting information for manuscript entitled:** **P****opulation genetic structure and adaptive differentiation of iron walnut *Juglans regia* subsp. *sigillata* in southwestern China**

**Yi-Wei Sun^1†^, Na Hou^2†^, Keith Woeste^3^, Chuchu Zhang^1^, Ming Yue^1,4^, Xiao-Ying Yuan^1^, Peng Zhao^1*^**

^1^Key Laboratory of Resource Biology and Biotechnology in Western China, Ministry of Education, College of Life Sciences, Northwest University, Xi’an, Shaanxi 710069, China

^2^Guizhou Academy of Forestry, Guiyang, China

^3^USDA Forest Service Hardwood Tree Improvement and Regeneration Center (HTIRC), Department of Forestry and Natural Resources, Purdue University, 715 West State Street, West Lafayette, Indiana, 47907, USA

^4^ Xi’an Botanical Garden of Shaanxi Province, Xi’an, Shaanxi 710061, China;

† These authors contributed equally to this work

***Correspondence author:** Peng Zhao

Email address: [pengzhao@nwu.edu.cn](mailto:pengzhao@nwu.edu.cn)

Tel: +86-029-88302411

Address: 229 Taibaibei Road, Xi’an, Shaanxi, China 710069

**Table S1** Chloroplast DNA sequence polymorphisms detected in five fragments of *Juglans sigillata*, identifying eight haplotypes (H1-H8).

| Loci | Sequence | Length | Gaps | M | S | P | Hd | π |
| --- | --- | --- | --- | --- | --- | --- | --- | --- |
| *trn*M*-*CAU | F: AAAGTCGTCTCCTTGAATCC  R: TGAACAACAAAGAGCGAGTA | 347 | 10 | 5 | 0 | 5 | 0.104 | 0.00098 |
| *trn*G-*trn*R | F: GCCTCACTTACTGGTATCTG  R: TGAACAACAAAGAGCGAGTA | 241 | 1 | 1 | 0 | 1 | 0.073 | 0.00016 |
| *JctrnS-trnR2* | F: TGAAAATTTGGGAAGAGGGA  R: TACCACTGAGTTAAAAGGGC | 319 | 1 | 1 | 0 | 1 | 0.073 | 0.00020 |
| *JctrnS-trnR6* | F: TCCGGACAAAATGGATGAAA  R: AAATATATAGGTGGTGGCCG | 367 | 12 | 1 | 0 | 1 | 0.042 | 0.00012 |
| *JrtrnF-ndhJ* | F: TTGGCTCAGTTTATCCGAAA  R: TCTGTTTTCTGGGTTTGGAA | 325 | 4 | 10 | 0 | 10 | 0.073 | 0.00210 |
| Average |  |  | 5.6 | 3.6 | 0 | 3.6 | 0.073 | 0.000712 |

Note: Length=PCR fragment length (bp); gaps=Sites with alignment gaps or missing data; M = Mutations=Total number of mutations; S=Singleton variable sites; P=Parsimony informative sites; Hd=Haplotype (gene) diversity; π=Nucleotide diversity

**Table** S2 A comparison of genetic diversity at 20 microsatellite loci in 36 ***J. sigillata*** populations.

| Locus | Na | *H*_O_ | *H*_S_ | *H*_T_ | *F*_ST_ | *G*’_ST_ |
| --- | --- | --- | --- | --- | --- | --- |
| JC8125 | 4 | 0.104 | 0.190 | 0.188 | 0.090 | 0.047 |
| JH89978 | 3 | 0.059 | 0.043 | 0.057 | 0.242 | 0.232 |
| JM5969 | 12 | 0.613 | 0.596 | 0.674 | 0.137 | 0.112 |
| JC7329 | 4 | 0.023 | 0.031 | 0.035 | 0.236 | 0.204 |
| JM61666 | 2 | 0.002 | 0.048 | 0.175 | 0.774 | 0.764 |
| JR4964 | 7 | 0.200 | 0.274 | 0.618 | 0.584 | 0.573 |
| JR4616 | 4 | 0.751 | 0.544 | 0.609 | 0.139 | 0.126 |
| JC5411 | 3 | 0.057 | 0.053 | 0.070 | 0.272 | 0.257 |
| JH42753 | 4 | 0.656 | 0.545 | 0.619 | 0.147 | 0.131 |
| JH86514 | 5 | 0.254 | 0.275 | 0.327 | 0.183 | 0.162 |
| JH91908 | 7 | 0.410 | 0.405 | 0.603 | 0.346 | 0.329 |
| JR3773 | 4 | 0.469 | 0.492 | 0.619 | 0.209 | 0.185 |
| JH84548 | 2 | 0.258 | 0.347 | 0.436 | 0.187 | 0.153 |
| JM78331 | 3 | 0.004 | 0.018 | 0.180 | 0.800 | 0.793 |
| JM68820 | 6 | 0.261 | 0.347 | 0.395 | 0.163 | 0.132 |
| JR6439 | 5 | 0.603 | 0.539 | 0.690 | 0.234 | 0.219 |
| JR3434 | 4 | 0.382 | 0.404 | 0.436 | 0.088 | 0.061 |
| JH6044 | 6 | 0.281 | 0.282 | 0.312 | 0.092 | 0.063 |
| JR6160 | 6 | 0.495 | 0.484 | 0.574 | 0.192 | 0.170 |
| JH2096 | 6 | 0.500 | 0.431 | 0.465 | 0.121 | 0.101 |
| Average | 4.85 | 0.319 | 0.317 | 0.404 | 0.262 | 0.241 |

Note: Number of Alleles (Na); observed heterozygosity over all populations (*H*o); gene diversity within populations (*h***_S_**); overall gene diversity (*H*_T_); among population differentiation (*F*_ST_) and standardized genetic differentiation (*G*’_ST_).

**Table S3** Rates of gene flow among three clusters as estimated using microsatellite data with program MIGTATE

|  | MIGRATE-N | | |  |
| --- | --- | --- | --- | --- |
|  | θ | *M* (m/μ) | |  |
|  |  | Cluster A → | Cluster B → | Cluster C → |
| Cluster A | 1.6202 | - | 2.0181 (0.025-0.995) | 2.5936 (0.025-0.995) |
| Cluster B | 1.1250 | 1.6789 (0.025-0.995) | - | 2.3369 (0.025-0.995) |
| Cluster C | 1.2060 | 3.0041 (0.025-0.995) | 5.6365 (0.025-0.995) | - |

**Table** S4 Posterior median estimate and 95 % highest posterior density interval (HPDI) for the demographic parameters in scenarios 4, based on the nuclear multi-locus microsatellite data of *Juglans sigillata.*

|  | Parameter | N1^a^ | N2^b^ | N3^c^ | t1 (generations) | t2 (generations) | μ | *P* |
| --- | --- | --- | --- | --- | --- | --- | --- | --- |
| Scenario 4 | Median | 4.37×10^5^ | 2.78×10^5^ | 3.13×10^5^ | 1.56×10^4^ | 6.25×10^4^ | 1.79×10^-6^ | 0.42 |
|  | Lower  bound | 9.33×10^4^ | 4.61×10^4^ | 5.48×10^4^ | 1.80×10^3^ | 1.66×10^4^ | 1.04×10^-6^ | 0.13 |
|  | Upper  bound | 9.26×10^5^ | 8.12×10^5^ | 8.46×10^5^ | 4.54×10^4^ | 9.78×10^4^ | 6.13×10^-6^ | 0.85 |

Note: The current population sizes of popA, popB, and popC were denoted as N1, N2 and N3 respectively.
